# A New Method to Evaluate Surface Defects with an Electromagnetic Acoustic Transducer

**DOI:** 10.3390/s150717420

**Published:** 2015-07-17

**Authors:** Kang Zhang, Pengxing Yi, Yahui Li, Bing Hui, Xuming Zhang

**Affiliations:** 1 School of Mechanical Science & Engineering, Huazhong University of Science & Technology, Wuhan 430074, China; E-Mails: zhangkang@hust.edu.cn (K.Z.); liyh20@163.com (Y.L.); Huibing@hust.edu.cn (B.H.); 2 School of Life Science & Technology, Huazhong University of Science & Technology, Wuhan 430074, China; E-Mail: zxmboshi@hust.edu.cn

**Keywords:** lift-off slope, surface defect, EMAT

## Abstract

Characterizing a surface defect is very crucial in non-destructive testing (NDT). We employ an electromagnetic acoustic transducer (EMAT) to detect the surface defect of a nonmagnetic material. An appropriate feature that can avoid the interference of the human factor is vital for evaluating the crack quantitatively. Moreover, it can also reduce the influence of other factors, such as the lift-off, during the testing. In this paper, we conduct experiments at various depths of surface cracks in an aluminum plate, and a new feature, lift-off slope (LOS), is put forward for the theoretical and experimental analyses of the lift-off effect on the receiving signals. Besides, by changing the lift-off between the receiving probe and the sample for testing, a new method is adopted to evaluate surface defects with the EMAT. Compared with other features, the theoretical and experimental results show that the feature lift-off slope has many advantages prior to the other features for evaluating the surface defect with the EMAT. This can reduce the lift-off effect of one probe. Meanwhile, it is not essential to measure the signal without defects.

## 1. Introduction

Through the electromagnetic acoustic transduction principle, electromagnetic acoustic transducers are widely used for thickness measurement and defect characterization without making physical contact with the test object [1,2]. For a defective sample, we not only detect whether there is a defect, but also the size of the defect. The EMATs generate and detect ultrasound in nonmagnetic or ferromagnetic materials depending on the Lorentz force mechanism, the magnetization force mechanism or the magnetostrictive force mechanism [3]. In this paper, we pay attention to the nonmagnetic materials for the Lorentz force mechanism, which is linear [4].

According to previous studies, many features are used to size a defect, such as ultrasonic time of flight (TOF), peak-to-peak amplitude, transmission coefficient, reflection coefficient and cut-off frequency [5,6,7,8,9,10,11,12]. The TOF is up to the wave velocity and the propagation distance, and there is no need to obtain a calibration to characterize the defect. However, the lift-off still affects this feature [5]. A small error of the measured TOF due to the variance of lift-off will lead to a negative estimate of the defect, especially for a shallow defect [5,13]. The other features are related to the structure of the probe and the exciting signal. Calibration is essential for estimating the defect. Moreover, the peak-to-peak amplitude is more sensitive to the lift-off, while the transmission coefficient, reflection coefficient and cut-off frequency are less. Based on the analysis of the lift-off effect on the receiving signal, a new feature, the “lift-off slope” (LOS), is put forward in this paper, and a new method is proposed for this feature in the EMAT testing. By changing the lift-off, we process the receiving signal and obtain the relationship between the lift-off and the crack. In [14], the authors used this method to detect a crack in pulsed eddy current (PEC) testing. However, the detection principles are different for the PEC and the EMAT. Besides, the means of signal processing in these two methods are different. For the PEC, the relationship between the receiving signal and the crack is linear, while this differs for the EMAT. With this method, the lift-off effect of one probe can be reduced, and a signal without a defect is not required during the characterization process.

In this paper, on the basis of the principle of the EMAT detection, we propose the feature “lift-off slope” to evaluate the surface defect within a metal part. The feasibility of LOS is verified by the experimental results. Meanwhile, we give a discussion of the proposed new evaluation method for surface defects.

## 2. Principle of the EMAT Detection

**Figure 1:**
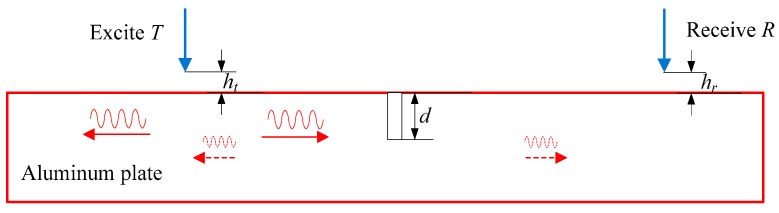
The schematic diagram of the EMAT detection.

The detection process of EMAT can be simplified as in Figure 1. The EMAT works in a pitch-catch mode. The transmitting probe and the receiving probe are located at different sides of the crack. In the transmitting module, the high alternating current pulsed through the transmitting coil induces eddy current within the sample skin depth. With the interaction of the eddy current and the external static field provided by the permanent magnet, the Lorentz force is yielded, as shown in Equation (1). According to Equation (2), the force generates elastic waves, which propagate in the opposite directions through the sample. The wave interacts with the crack, a part of the wave transmits through the crack and a part of the wave reflects from the crack. In this paper, we only pay attention to the transmitting wave. The receiving probe is far away from the crack to avoid the near-field enhancement. In the receiving module, the particle velocity induced by the ultrasonic motion interacts with the bias field to yield current density in the sample. In turn, a voltage induced in the receiving coil is produced by the time varying magnetic field generated by the current density. When the defect is changed, the voltage signal will change. By analyzing the relationship between the voltage and the defect, we can characterize the defect with the features extracted from the signal.

(1) F=J×B

(2) $$\text{ρ}\frac{\partial^{2}u}{\partial t^{2}}=\nabla •T+F$$

where *B* is the magnetic flux intensity, *J* is current density, σ is the electrical conductivity, *ρ* is the mass density, *u* is the elastic deformation, *F* is the force per volume and *T* is the elastic stress tensor.

## 3. Theoretical Model

**Figure 2:**
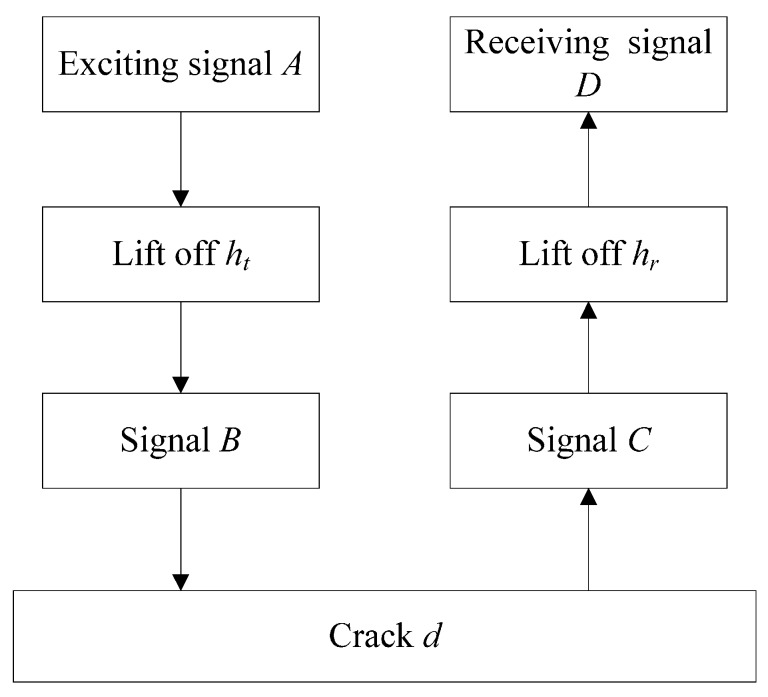
The interaction of the signals.

According to the description of the testing process of the EMAT, the interaction of the signals can be simplified as in Figure 2. When the pulsed current is sent through the coil, the exciting signal *A* is generated in the probe. There is a distance between the transmitting probe and the sample, namely the lift-off *h_t_*. The signal *A* transforms into signal *B*. Signal *C* is obtained after the wave is blocked by the surface crack *d*. Through the lift-off *h_r_* between the receiving probe and the sample, the receiving signal *D* is picked up in the coil. The above processes can be expressed in Equations (3)–(5).

(3) $$B=f_{1}(h_{t})A$$

(4) $$C=f_{2}(d)B$$

(5) $$D=f_{3}(h_{r})C$$

where *h_t_* is the distance between the transmitting EMAT probe and the sample, *h_r_* is the distance between the receiving EMAT probe and the sample and *d* is the depth of the surface crack.

Then, the received signal *D* is shown as Equation (6):

(6) $$D=f_{1}(h_{t})f_{2}(d)f_{3}(h_{r})A$$

According to the [15], the relationship of the received voltage and the exciting current is shown in Equation (7). For the noncontact case, the lift-off effects of the transmitting probe and the receiving probe are processed by multiplying the factors *C_R_* and *C_T_*, respectively. This implies that the received signal can be expressed in the form of Equation (6). Compared with Equation (6), it can be seen that the function *f*_1_ and the function *f*_3_ correspond to the factors *C_T_* and *C_R_*, respectively. The functions of the *C_T_* and the *C_R_* are exponential [15]. Meanwhile, the exponential form is also verified in [16]. Therefore, the form of the functions *f_1_* and *f_3_* is exponential.

(7) $$\left|\frac{V_{R}}{I_{T}}\right|=2w^{2}B_{0R}B_{0T}N_{R}N_{T}C_{R}C_{T}W{\left|\frac{\sin\theta }{Y_{M1}^{1/2}}-e^{-j\phi }\frac{\cos\theta }{Y_{M3}^{1/2}}\right|}_{R}•{\left|\frac{\sin\theta }{Y_{M1}^{1/2}}-e^{-j\phi }\frac{\cos\theta }{Y_{M3}^{1/2}}\right|}_{T}$$

where *w* is the frequency of the signal, *B_0_* is the magnetic flux density, *N* is the turns of the coil, *W* is the length of the wire, *θ* is the angle at which the magnetic field intersects with the axis, *Y_M_* is the mode admittance and the subscripts 1 and 3 are introduced to differentiate between the x axis and z axis.

According to Equation (6), the peak-to-peak amplitude of the receiving signal *D* is a function of the crack depth *d*. When the depth of the crack increases, the peak-to-peak amplitude decreases. Besides, if the lift-off *h_t_* or *h_r_* varies, an overestimate or underestimate of the depth will be obtained due to the negative peak-to-peak amplitude [17].

The expression of the transmission coefficient is given by Equation (8). Under the same lift-off *h_t_* and *h_r_*, the transmission coefficient *Trans* is calculated such that the signal *D* with a defect is divided by the signal *D_0_* without a defect.

(8) $$Trans=\frac{D}{D_{0}}=\frac{f_{1}(h_{t})f_{2}(d)f_{3}(h_{r})}{f_{1}(h_{t})f_{2}(0)f_{3}(h_{r})}=\frac{f_{2}(d)}{f_{2}(0)}$$

Equation (6) illustrates that the transmission coefficient *Trans* is only the function of the parameter *d*. The functions *f_1_* and *f_3_* do not exert an effect on the transmission coefficient. Nevertheless, we need to measure both the signal without a defect and the signal with a defect at the same lift-off, which will make the testing complicated.

In accordance with the above expressions of the features, we put forward a new feature, LOS, which balances the lift-off and the measured signal. As we all know that when *h_t_* increases, the value of *f_1_*(*h_t_*) decreases, thus *f_1_* is a monotonic decreasing function. Meanwhile, both *f_2_* and *f_3_* are monotonic decreasing functions.

Supposing the *f_3_*(*h_r_*) is the independent variable, then Equation (6) is the function of the (*f_3_*(*h_r_*), *D*). We use the value *K* to represent the slope of the function (*f_3_*(*h_r_*), *D*). The slope *K* can be expressed by the Equation (9).

(9) $$K=f_{1}(h_{t})f_{2}(d)A$$

If the lift-off *h_t_* is kept as a constant and the exciting signal *A* is kept unchanged, *K* is the monotonic decreasing function of the crack depth *d*. In other words,

If:

(10) $$d_{1}<d_{2}<d_{3}<...<d_{n}$$

Then:

(11) $$K_{1}>K_{2}>K_{3}>...>K_{n}$$

Therefore, the curve of the dependent variable *D* and the independent variable *f_3_*(*h_r_*) can be gotten by changing the lift-off *h_r_*. The defect can be calculated by measuring the slope *K* of the curve. It can be seen that the slope *K* is not relevant to the parameter *h_r_* and the signal without a defect.

## 4. Experiment Details

**Figure 3:**
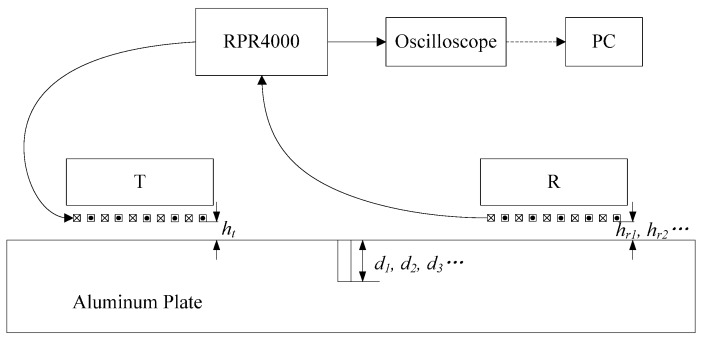
The schematic diagram of the experiment.

Figure 3 illustrates the schematic diagram of the experiment. As shown in Figure 3, the RPR4000 is a high power pulsed generator and receiver. A high power pulsed current is sent through the transmitting probe T, and the wave propagating in the aluminum plate interacts with the crack. At first, the receiving probe R, whose structure is the same as the transmitting probe T, acquires the signal and sends the signal to the RPR4000. Then, the signal is filtered and amplified by the RPR4000. The processed data are saved and displayed in the oscilloscope. Finally, the data are processed on the PC.

**Figure 4:**
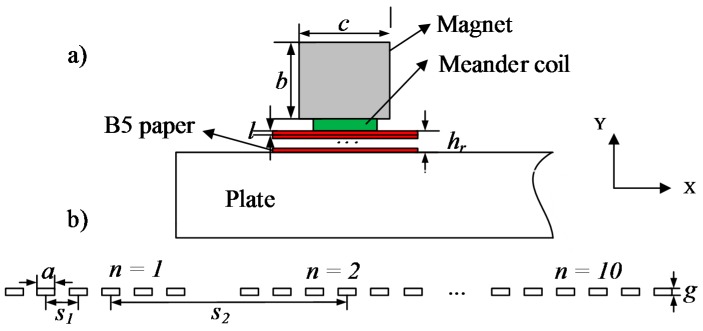
(**a**) The structure of the probe; (**b**) the schematic diagram of the coil.

Figure 4 shows the geometric parameters of the probes. The probe consists of the magnet and the meander coil, which is made of printed circuit board. The x-direction length *c*, the y-direction length *b* and the z-direction length of the magnet are 40 mm. The remnant magnetic flux intensity is 1.21 T. Figure 4b shows the details of the meander coil. The thickness of the printed circuit board is 0.6 mm. The coil includes 10 bends, and every bend consists of 6 conductor wires. The width of the conductor wire *a* is 0.15 mm. The interval *s_1_* of the adjacent conductors is 0.3 mm. The height *g* of the coil is 0.035 mm. The interval *s_2_* of the adjacent bends is 3.0 mm.

A three-cycle sine burst is sent through the meander coil. The peak-to-peak voltage of the exciting signal is 350 V, and the frequency is 500 kHz. The material of the samples is 6061 aluminum alloy, and the dimension is 500 × 250 × 60 mm. At the center of the samples, the slots were machined vertically on the surface with a depth ranging from 0 mm to 5 mm and a width of 1 mm. In order to eliminate the near-field effect, both the transmitting probe and the receiving probe are 150 mm away from the crack [11,18]. According to the structure of the probe and the thickness of the aluminum plate, the wave generated in the experiment is the Rayleigh wave.

In this experiment, we kept the exciting signal and the transmitting probe unchanged. The value of the lift-off *h_t_* is 0 mm. At a certain depth *d_i_*, we measured the signals at lift-off *h_r1_*, *h_r2_* and *h_rn_*. The lift-off *h_r_* between the coil and the plate was achieved by inserting B5 papers between the probe and the aluminum plate [10]. The thickness *l* of a sheet of B5 paper is 0.1 mm. This can assure the accuracy of the lift-off and keep the lift-off stable in the experiment.

## 5. Experimental Results

### 5.1. The Lift-Off Effect

**Figure 5:**
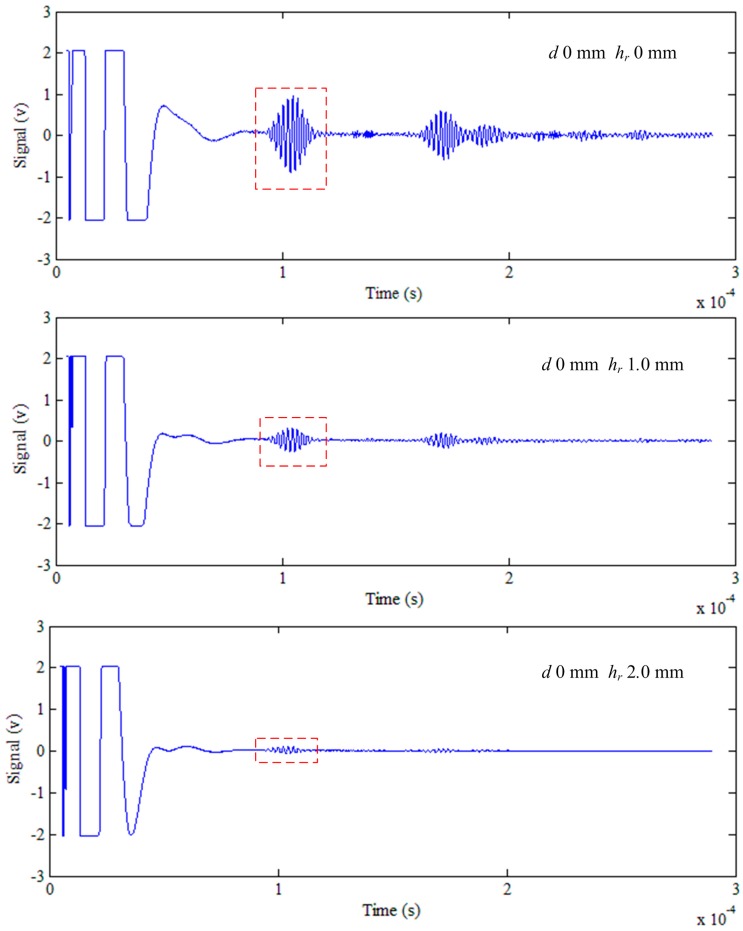
The signal at lift-off of 0 mm, 1.0 mm and 2.0 mm of a defect depth of 0 mm.

**Figure 6:**
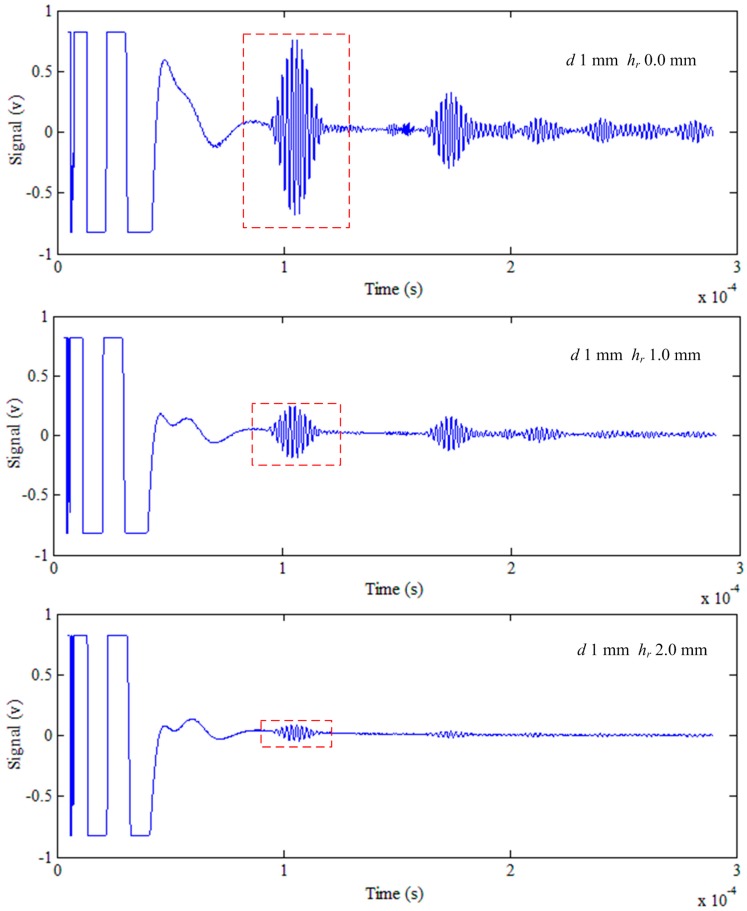
The signal at lift-off of 0 mm, 1.0 mm and 2.0 mm of a defect depth of 1 mm.

**Figure 7:**
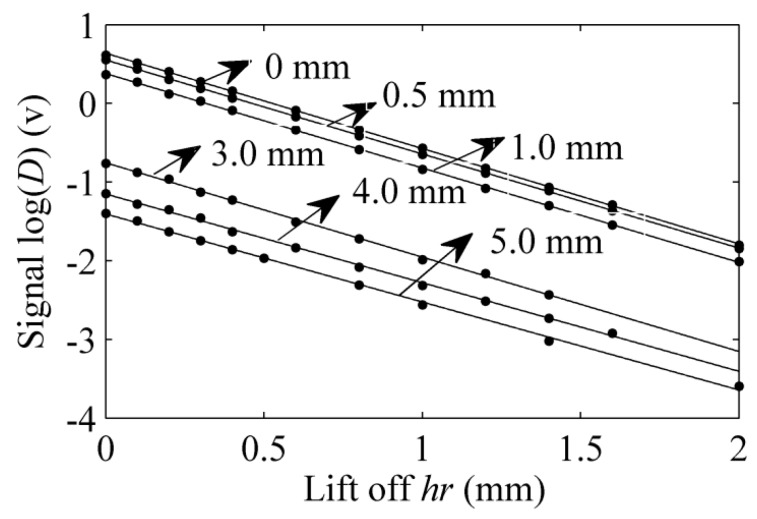
The relationship of *h_r_* and log(*D*).

When the lift-off increases, the signal decreases. Figure 5 and Figure 6 show the signal at different lift-offs of defect depths of 0 mm and 1.0 mm, respectively. The different lift-offs *h_r_* and the corresponding peak-to-peak logarithmic signals log(*D*) are fitted in the least squares method with MATLAB software. The relationship between *h_r_* and log(*D*) is plotted in Figure 7. Table 1 shows the slope of the fitting line and the correlation coefficient. With the increasing of the lift-off *h_r_*, the signal log(*D*) decreases. As a result, the correlation coefficients are negative. The minimum absolute value of the correlation coefficients is 0.9992, which is bigger than 0.99. This implies that the linearity of the lift-off *h_r_* and the logarithmic signal log(*D*) is very high. Therefore, it is reasonable to regard the relationship between *h_r_* and *D* as an exponential distribution, and this is verified in [15].

**Table 1:** The slope of the curve (*h_r_*, log(*D*)) and the correlation coefficient.

Defect Depth (mm)	Slope T	Correlation Coefficient
0	−1.1215	−0.9999
0.5	−1.1958	−1.0000
1.0	−1.2004	−0.9999
3.0	−1.1992	−0.9992
4.0	−1.1269	−0.9992
5.0	−1.1186	−0.9993

**Figure 8:**
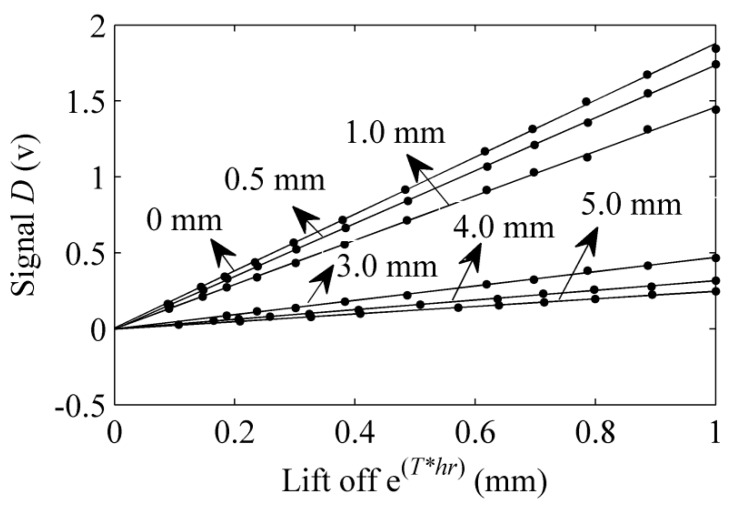
The relationship of e^(T*hr)^ and signal *D.*

The same as the above method, the data (e^(T*hr)^, *D*), illustrated in Figure 8, are fitted linearly. Besides, the slope of the fitting line and the correlation coefficient are shown in Table 2. The dependent variable *D* and the independent variable e^(T*hr)^ are positively correlated, and the minimum value of the correlation coefficient is bigger than 0.99. In contrast with Equation (9), the function *f_3_*(*h_r_*) is e^(T*hr)^, and the slope *K* is the feature that we want. As is shown in Figure 8 and Table 2, the slope *K* decreases monotonically when the depth *d* of the crack increases.

**Table 2:** The slope of the curve (e^(T*hr)^, *D*) and the correlation coefficient.

Defect Depth(mm)	Slope K	Correlation Coefficient
0	1.8728	0.9997
0.5	1.7394	0.9999
1.0	1.4605	0.9997
3.0	0.4721	0.9991
4.0	0.3190	0.9990
5.0	0.2497	0.9996

### 5.2. A New Method with LOS to Characterize the Defect

#### 5.2.1. The Process of the New Method

**Figure 9:**
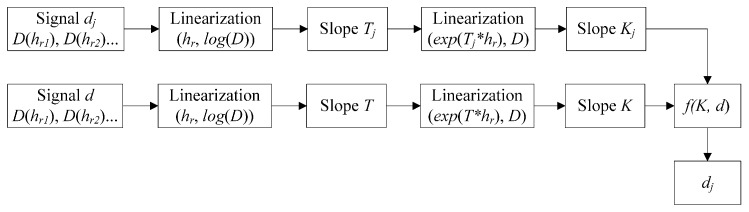
The process of the new method with the lift-off slope (LOS) for characterizing the defect.

Based on the above results, a new method is proposed to characterize the defect quantitatively through the EMAT. The method is described in Figure 9. We measure the signals at different lift-offs under different depths. Firstly, the signals are linearized to calculate the slope *T*, and the exponents e^(T*hr)^, namely the independent variables *f_3_*, are obtained. Then, the data (e^(T*hr)^, *D*) are linearized to get the feature, LOS *K*, corresponding to the depth *d*. On the basis of the fitting to the data (*K*, *d*), a calibration *f*(*K*,*d*) can be obtained. The same as the method for an unknown depth *d_j_* of the crack, we can get the LOS *K_j_*. Comparing this value *K_j_* with the calibration *f*(*K*,*d*), the depth *d_j_* can be found.

#### 5.2.2. The Accuracy of the New Method to Evaluate an Unknown Defect

**Figure 10:**
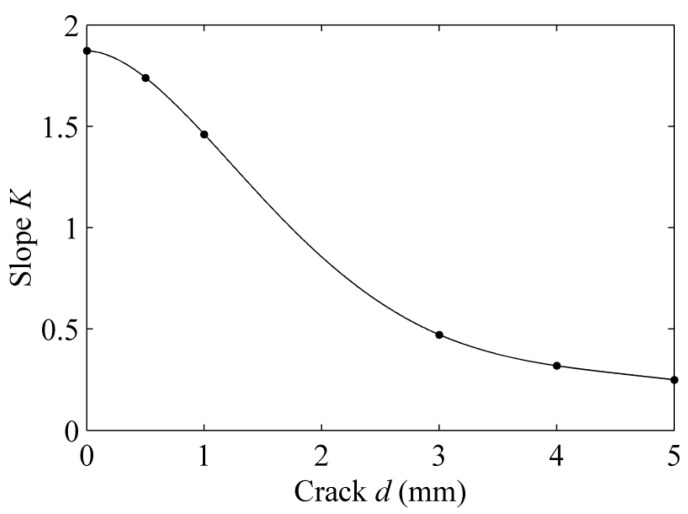
The calibration of slope *K* and crack *d*.

A fifth degree polynomial fit is applied to handle the data shown in Table 2. The correlation coefficient is −0.9748. Figure 10 depicts the relationship of the slope *K* and the depth of crack *d*. The fitting function is expressed by Equation (12).

(12) $$K=0.0021d^{5}-0.0379d^{4}+0.244d^{3}-0.5946d^{2}-0.0259d+1.8728$$

We detected a new sample with a crack whose depth is 1.3 mm. Through the aforementioned method, we processed the received signal, and the slope *K_j_* is 1.3348. According to the calibration, the inferred depth *d_j_* is 1.2 mm. The relative error between the actual depth and the inferred depth is 7.7%. The results show that we can evaluate the crack precisely with the feature lift-off slope.

#### 5.2.3. The Sensitivity of Different Features to the Lift-Off

During the detection process, if we make a mistake about the actual distance between the receiving probe and the plate, an inaccurate evaluation of the depth of the crack with different features will occur. For example, the actual lift-off is 0.3 mm; however, because of a measurement error, we mistake the lift-off for 0.2 mm. For other lift-offs, we assume that the measurement lift-offs are right. Therefore, different features are adopted to calculate the depth with the calibration at lift-off of 0.2 mm. This means that we may make a negative evaluation of the depth if the calibration at 0.2 mm is not the same as the calibration at 0.3 mm. The absolute errors and the relative errors between the calculated depth and the real depth are illustrated in Table 3.

The data listed in Table 3 show that the relative errors of the depth are small when the transmission coefficient and the lift-off slope are chosen, while they are big for peak-to-peak amplitude. At a crack depth of 0.5 mm, the relative errors are a little bigger. This may be caused by the fact that the denominator “0.5 mm” is small. In general, the transmission coefficient and the lift-off slope are less sensitive to the lift-off. However, for the feature transmission coefficient, we need to measure the signal without a defect, and the lift-off should be the same as the one with a defect. Moreover, with the feature lift-off slope, we do not need to measure the signal without a defect.

**Table 3:** The absolute error and the relative error of the calculated depth caused by inaccurate lift-off.

Defect Depth (mm)	Peak-to-Peak Amplitude (v)			Transmission Coefficient			Lift-off Slope K		
	Calculated Depth (mm)	Absolute Error (mm)	Relative Error	Calculated Depth (mm)	Absolute Error (mm)	Relative Error	Calculated Depth (mm)	Absolute Error (mm)	Relative Error
0	0.601	0.601	---	0	0	---	0.269	0.269	---
0.5	0.833	0.333	66.6%	0.45	−0.05	10.0%	0.577	0.077	15.4%
1.0	1.203	0.203	20.3%	0.915	−0.085	8.5%	1.041	0.041	4.1%
3.0	3.367	0.367	12.2%	3.079	0.079	2.6%	3.057	0.057	1.9%
4.0	4.367	0.367	9.2%	3.914	−0.086	3.0%	4.077	0.077	1.9%
5.0	5.569	0.569	11.4%	4.941	−0.059	1.2%	5.083	0.083	1.7%

## 6. Conclusions

In view of the interactions of the signals in the EMAT testing, we proposed the expressions of the features of the peak-to-peak amplitude and the transmission coefficient. Meanwhile, we can have an in-depth understanding of the effect of the lift-off effect and the crack on the features. On the basis of the expressions of the features, a new feature, lift-off slope, was defined. We proposed a theoretical model to investigate the feasibility of this feature to evaluate the surface defects, which was also verified by the experiments.

Taking the lift-off slope into account, a new method to evaluate the surface defect was proposed in the EMAT testing, as well. By analyzing the relationship between the lift-off slope and the depth of the crack, we can obtain the unknown depth of the defect through the calibration. Compared with the feature peak-to-peak amplitude, the LOS can reduce the lift-off effect of the receiving probe. Besides, compared with the transmission coefficient, the defect can be evaluated without measuring the signal without a defect through the LOS. It can be known that the feature LOS achieves a balance between the peak-to-peak amplitude and the transmission coefficient.

The theoretical and experimental results show that we can evaluate the defect with only two different lift-offs. However, this may lead to enormous errors. If the signal of one lift-off is inaccurate, the slope will be greatly changed. This will lead to a negative evaluation of the depth of the crack. Therefore, we need to measure the signal at different lift-offs, as many as possible. This means that we cannot save time with this method during the testing process.

Surely, the method cannot reduce both the lift-off effects of the transmitting probe and the receiving probe. In this paper, we limited ourselves to analyzing the lift-off effect of the receiving probe. Compared with the receiving probe, the variation of the lift-off of the transmitting probe will lead to the change of the exciting signals [19]. That may cause an inaccurate result. In further study, we should keep the exciting signals unchanged and then investigate the lift-off effect of the transmitting probe. Besides, the Lorentz mechanism is linear while the magnetization force mechanism and the magnetostrictive force mechanism are not. In that way, the function *f_3_* may not be exponential. How to express the feature lift-off slope is a major problem to be studied for the ferromagnetic material. Moreover, the crack location may affect the received signal. Additionally, we will study the effect of the crack location on the feature.
